# Essential role of glucokinase in the protection of pancreatic β cells to the glucose energetic status

**DOI:** 10.1038/s41420-019-0219-x

**Published:** 2019-09-30

**Authors:** Patricia Marqués, Anne Kamitz, Alberto Bartolomé, Jesús Burillo, Helena Martínez, Beatriz Jiménez, María Fernández-Rhodes, Carlos Guillén, Manuel Benito

**Affiliations:** 1 Complutense University, Madrid, Spain; 2 Naomy Berrie Diabetes Center, New York, NY USA; 3 Centro de Investigación Biomédica en Red (CIBER) de Diabetes y Enfermedades Metabólicas Asociadas (CIBERDEM), Madrid, Spain

**Keywords:** Metabolic disorders, Mechanisms of disease

## Abstract

Energy sensing is indispensable to balance anabolic and catabolic processes for the maintenance of cell viability. Pancreatic β cells are especially relevant because of their involvement in the coordination of insulin secretion when glucose concentration arises in the local milieu. In this work, we uncover the increased susceptibility of pancreatic β cells to cell death in response to different energy stressors. Upon glucose decline, from 25 to 5 mM, caused by stimulation with either 2-deoxyglucose or metformin, only pancreatic β cells showed an increase in cell death. Very interestingly, when we transfected either mouse insulinoma cell or human embryo kidney cells with a phospho-mutant form of B cell lymphoma 2 associated agonist of cell death at serine 155 (BAD S155D), an increase in the pro-survival factor B cell lymphoma 2 was detected in pancreatic β cells and not in human embryonic kidney cells in the presence of the energetic stressors. This data suggests that the protective capacity of this mutant form is only present in cells that present glucokinase. In contrast, upon hyperactivation of mechanistic target of rapamycin complex 1 signaling by knocking-down tuberous sclerosis complex protein, we observed increased susceptibility to cell death in response to energy stress in both pancreatic and non-pancreatic β cells. Therefore, mechanistic target of rapamycin complex 1 signaling presents a dual effect on cell viability. On the one hand, a chronic inhibition of mechanistic target of rapamycin complex 1 activity in response to the energy status is deleterious for pancreatic β cells, being attenuated by the overexpression of B cell lymphoma 2 associated agonist of cell death S155D. On the other hand, mechanistic target of rapamycin complex 1 hyperactivity provokes a susceptibility to energetic stress-induced cell death. Taken together, these results may open potential implications for the use of glucokinase activators or mechanistic target of rapamycin complex 1 modulators for the maintenance of pancreatic β cells for longer periods of time avoiding its loss in different pathologies such as type 2 diabetes mellitus.

## Introduction

The mechanistic target of rapamycin (MTOR) is a serine–threonine protein kinase that belongs to the PI3K-related kinase family^1^. It regulates eukaryotic cell growth and metabolism in response to stimuli, including nutrients and growth factors comprising the catalytic subunit of two complexes: mTOR complex 1 (MTORC1) and mTOR complex 2 (MTORC2). MTORC1 is defined by its catalytic subunit (mTOR) and some exclusive proteins: RPTOR (regulatory protein associated with mTOR), mLST8 (mammalian lethal with Sec13 protein 8, also known as GβL), proline-rich AKT substrate 40 kDa (PRAS40), and DEP-domain-containing mTOR-interacting protein (Deptor)^2^. RPTOR facilitates substrate recruitment through binding to a TOR signaling (TOS) motif^3^ and is necessary to the subcellular localization of the complex. The substrates of MTORC1 are S6 kinase 1 (S6K1) and 4E-BP1 (elF4E binding protein 1), which control protein synthesis and ribosomal biogenesis. MTORC1 binding to the active form of Ras homolog—enriched in brain (RHEB) (RHEB-GTP) and localized on lysosomal and endosomal membranes, is essential for the activation of MTORC1. RHEB activity is regulated by the tuberous sclerosis complex (TSC), which is formed of tuberous sclerosis complex 1 (TSC1 or hamartin), tuberous sclerosis complex 2 (TSC2 or tuberin) and Tre2-Bub2-Cdc16-1 domain family member 7 (TBC1D7)^4^. TSC2 presents a GTPase activating protein (GAP) domain, which enables RHEB to inhibit MTORC1 and it is recruited to the surface of the lysosome in response to multiple stress signals: low energy, hypoxia, amino-acid starvation, hyperosmotic stress, and others^5–7^.

Glucokinase (GK) is a glycolytic enzyme present in β cells and hepatocytes, associated to the BCL-2 family pro-apoptotic protein BAD at the mitochondrial membrane^8,9^. GK is regulated by multiple mechanisms, including its association with and activation by BAD. When BAD protein is phosphorylated on Ser 155, GK is capable of stimulating β cells to secrete insulin and improve their function and survival^10^.

The AMP-activated protein kinase (AMPK) is a heterotrimeric serine–threonine kinase composed of a catalytic α domain and βγ regulatory domains^11^ that plays a critical role in regulating cellular energy homeostasis. It is known that AMPK directly phosphorylates RAPTOR at Ser 792 and Ser 722, suppressing MTORC1 activity under different stress situations such as low levels of ATP, acting as a metabolic sensor of cellular energy status^12^. AMPK can phosphorylate TSC2 on Thr 1227 and Ser 1345 for MTORC1 downregulation^13^. Recent papers indicate that AMPK activation can occur by the interaction of fructose-1,6-bisphosphate (FBP), which is a glycolytic intermediate and interacting with the aldolase. This complex (FBP-aldolase) could interact with the vacuolar-ATPase (v-ATPase) on the lysosomal membrane. When glucose deprivation occurs, FBP diminishes, and there is an interaction between v-ATPase and aldolase, favoring the interaction with PRKAA-AXIN. This new interaction facilitates the phosphorylation of AMPK by the kinase LKB1^14,15^. Although AMPK activation is necessary for inhibiting MTORC1 signaling, it is known that pancreatic β cells are especially prone to die by apoptosis after a chronic stimulation of AMPK^11,16,17^.

In this paper, we have determined the importance of the cell energetic maintenance, especially in pancreatic β cells compared to other cell types. Based on the mechanisms involved in response to energetic stress between pancreatic and non-pancreatic β cells, we propose that GK activators or inducers of TSC2 activity could allow β cells to sense the lack of glucose and to adapt to energetic stress, avoiding apoptosis.

## Results

### Pancreatic β cells are more susceptible to energy deprivation than other cell lines

**Fig. 1:**
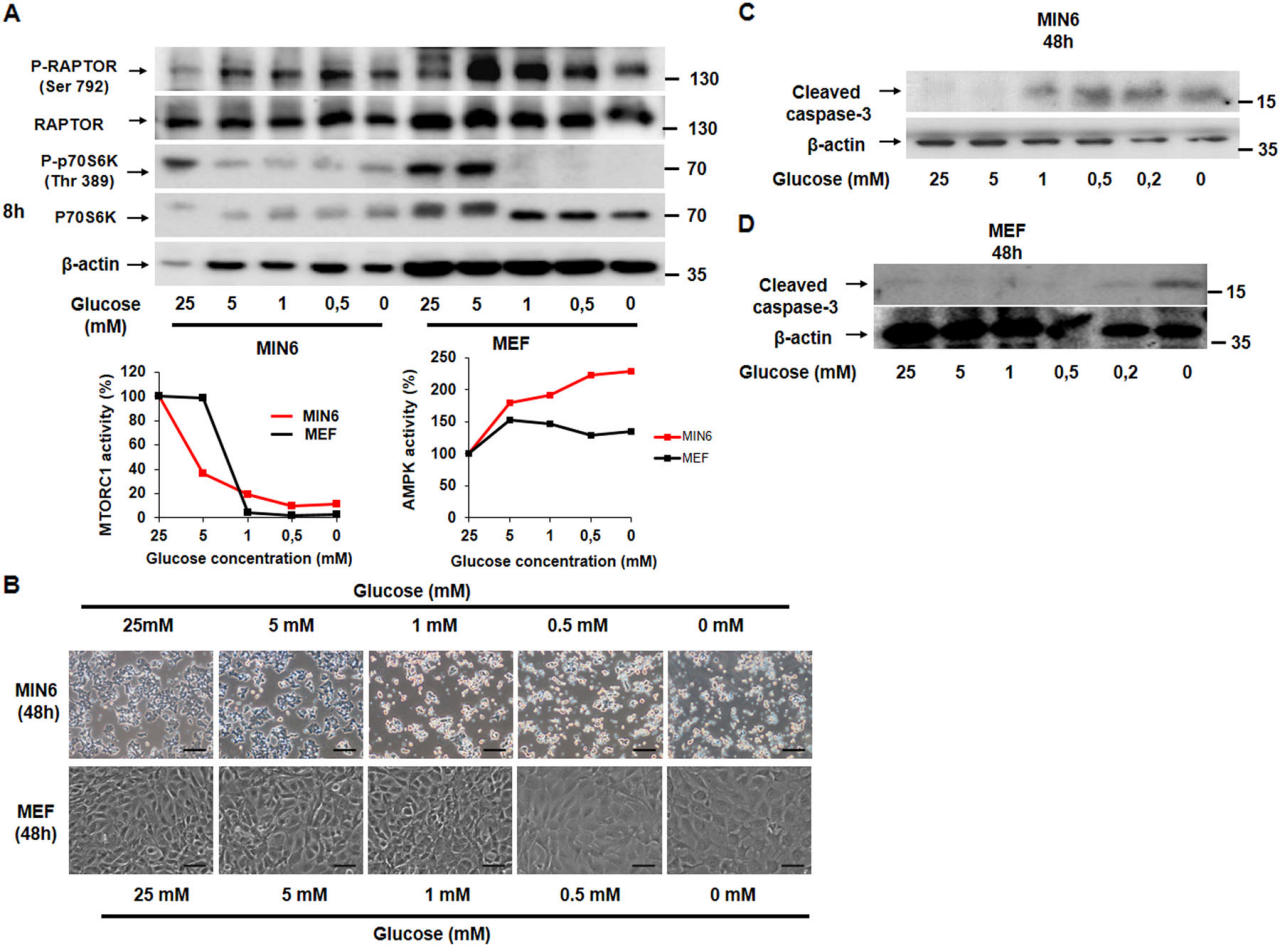
Pancreatic β cells are more susceptible to glucose deprivation than MEF cells. **a** Representative western blot analysis (*n* = 3) of MIN6 and MEF cells challenged to glucose deprivation (from 25 mM to no glucose). In the plot is showing the densitometry analysis of the image (MTORC1 activity and AMPK activity). **b** Representative phase-contrast images comparing MIN6 and MEF in response to glucose deprivation. Scale bar, 40 µm. **c** Representative western blot analysis showing the cleaved caspase-3 protein levels in a dose-response of glucose deprivation in MIN6 cells. **d** Representative western blot showing cleaved caspase-3 protein levels in a dose-response of glucose deprivation in MEF cells

**Fig. 2:**
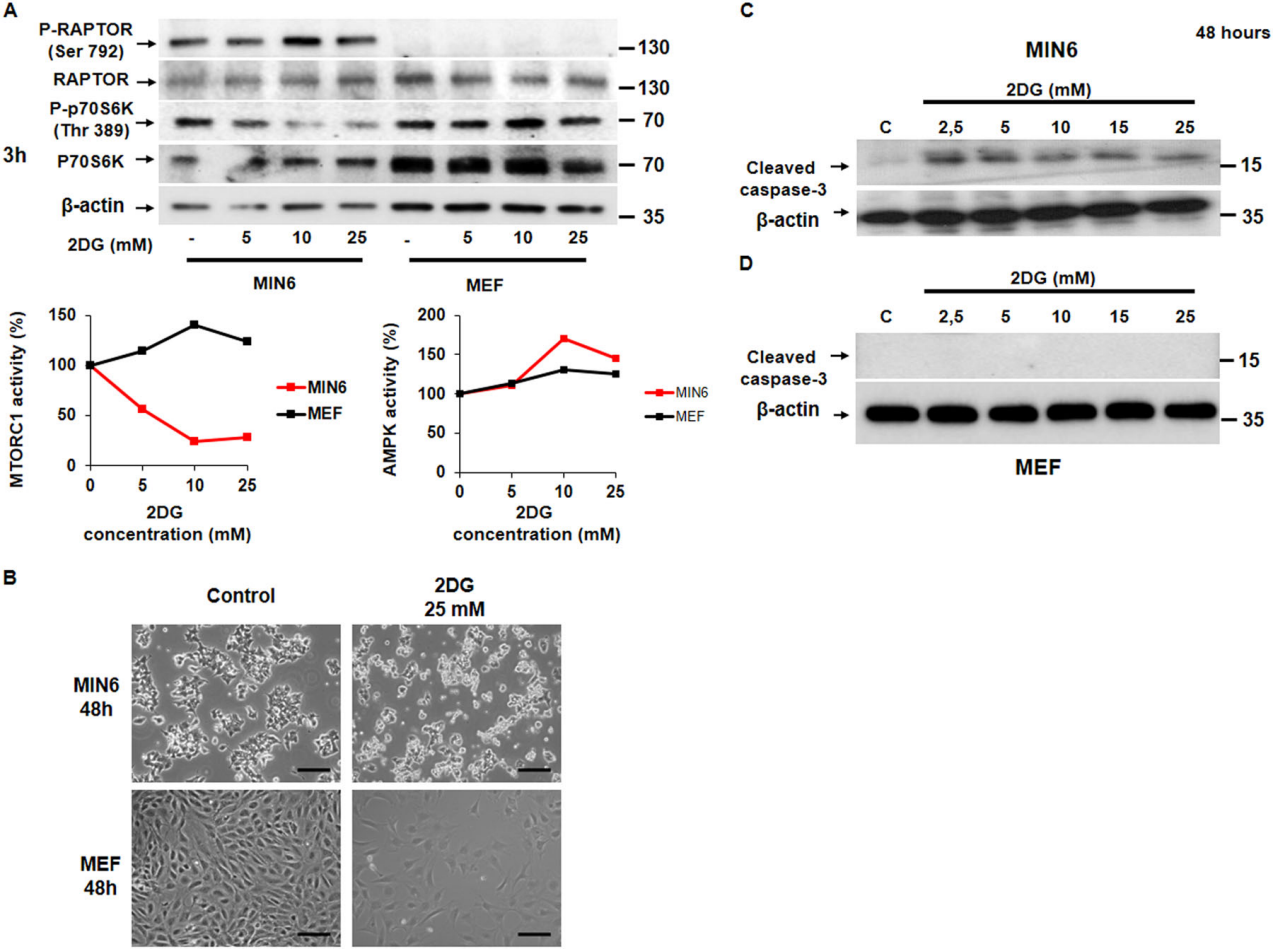
Pancreatic β cells are more susceptible to 2DG than MEF cells. **a** Representative western blot analysis (*n* = 3) of MIN6 and MEF cells subjected to a 2DG dose-response (from 5 mM to 25 mM in the presence of 25 mM of glucose). The plot indicates the densitometric analysis of the image (MTORC1 Activity and AMPK activity). **b** Representative phase-contrast images comparing MIN6 and MEF in response to the highest dose of 2DG (25 mM). Scale bar, 40 µm. **c** Representative western blot analysis showing the cleaved capase-3 protein levels in a dose-response of 2DG in MIN6 cells. **d** Representative Western blot showing cleaved caspase-3 protein levels in a dose-response of 2DG in MEF cells

**Fig. 3:**
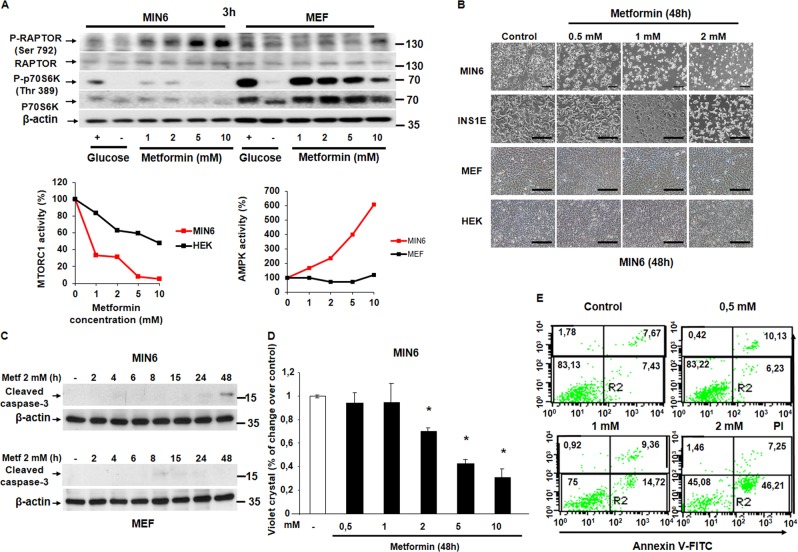
Pancreatic β cells are more susceptible to metformin than MEF cells. **a** Representative western blot analysis (*n* = 3) of MIN6 and MEF cells challenged to a metformin dose-response (1–10 mM in the presence of 25 mM of glucose). In the plot is represented the densitometric analysis of the image. **b** Representative phase contrast images comparing pancreatic β cells such as MIN6 or INS1E cells with non-pancreatic β cells such as MEF or HEK in response to different doses of metformin (0, 5–1–2 mM). Scale bar, 40 µm. **c** Representative western blot analysis showing the cleaved caspase-3 protein levels in a time-course of metformin at 2 mM in MIN6. **d** Representative western blot analysis showing the cleaved caspase-3 protein levels in a time-course of metformin at 2 mM in MEF cells. **e** Representative quantification of violet crystal analysis of the number of viable cells after treatment with dose-response of metformin for 48 h in MIN6. The percentage of change over control represents the mean ± SD. *P < 0.05. **f** Flow cytometry analysis of the percentage of apoptotic MIN6 cells after a dose-response of metformin treatment using Annexin-V and PI incorporation. PI stands for propidium iodide

We first measured the importance of energy supply for pancreatic β cell viability. We tested the effect of different energetic stressors such as glucose deprivation, 2DG, and metformin in both MTORC1 activity and cell survival. 2DG is a glucose analog and cannot be metabolized generating glycolysis blockade, and metformin is an inhibitor of the electron respiratory chain complex I. All the treatments blocked MTORC1 signaling very efficiently in MIN6 and less significantly in MEF cells (Figs. 1a, 2a, and 3a). We observed a reduction in MTORC1 signaling when dropping glucose concentration from 25 to 5 mM with a parallel increase in AMPK signaling by RAPTOR phosphorylation. In contrast, in fibroblasts, the inhibition of MTORC1 activity occurred at a glucose concentration below 5 mM, although AMPK activation was very efficient in all the conditions of glucose concentration (Fig. 1a). In response to 2DG, only MIN6 cells were capable to down-regulate MTORC1 signaling with a concomitant increase in AMPK signaling (Fig. 2a). Importantly, the effective dose of metformin for a complete MTORC1 inhibition was nearby 1 mM in MIN6 vs. 10 mM in MEF (Fig. 3a). Then, we analyzed the consequences on cell survival, by testing whether or not the energetic stressors were able to induce cell death. We observed by phase-contrast microscopy a reduction in the number of cells in response to all the energetic stressors used in MIN6 compared with MEF (Figs. 1b, 2b, and 3b). Glucose deprivation as well as 2DG increased cleaved caspase-3 protein levels after 48 h in a dose-dependent manner in MIN6 (Figs. 1c, 2c) but not in MEF (Figs. 1d, 2d), although a slight increase in annexin-V-propidium iodide staining was observed (Supplementary Fig. 1). Similarly, metformin at 2 mM induced cleaved caspase-3 protein levels in a time-dependent manner in MIN6 (Fig. 3c) but not in MEF cells (Fig. 3d). The effect of metformin decreasing cell survival was dose-dependent, by the analysis of the remaining attached cells by violet crystal in MIN6 cells (Fig. 3e). In addition, annexin-V-propidium iodide staining indicated that metformin was able to induce early apoptotic events (Fig. 3f). Using flow cytometry analysis, we further corroborated that metformin is capable of stimulating cell death by analyzing cells in sub-G1 cell phase in a dose and time-dependent manner in MIN6 (Supplementary Fig. 2). This deleterious effect of metformin was confirmed in other pancreatic β cell types such as INS1E cells and hepatocytes, in the presence of GK (Supplementary Fig. 3). Combined together, these results clearly indicate that pancreatic β cells are more susceptible to cell death in response to energy deprivation than other cell types.

### TSC2 is an essential regulator of mTORC1 signaling in response to energy stress

**Fig. 4:**
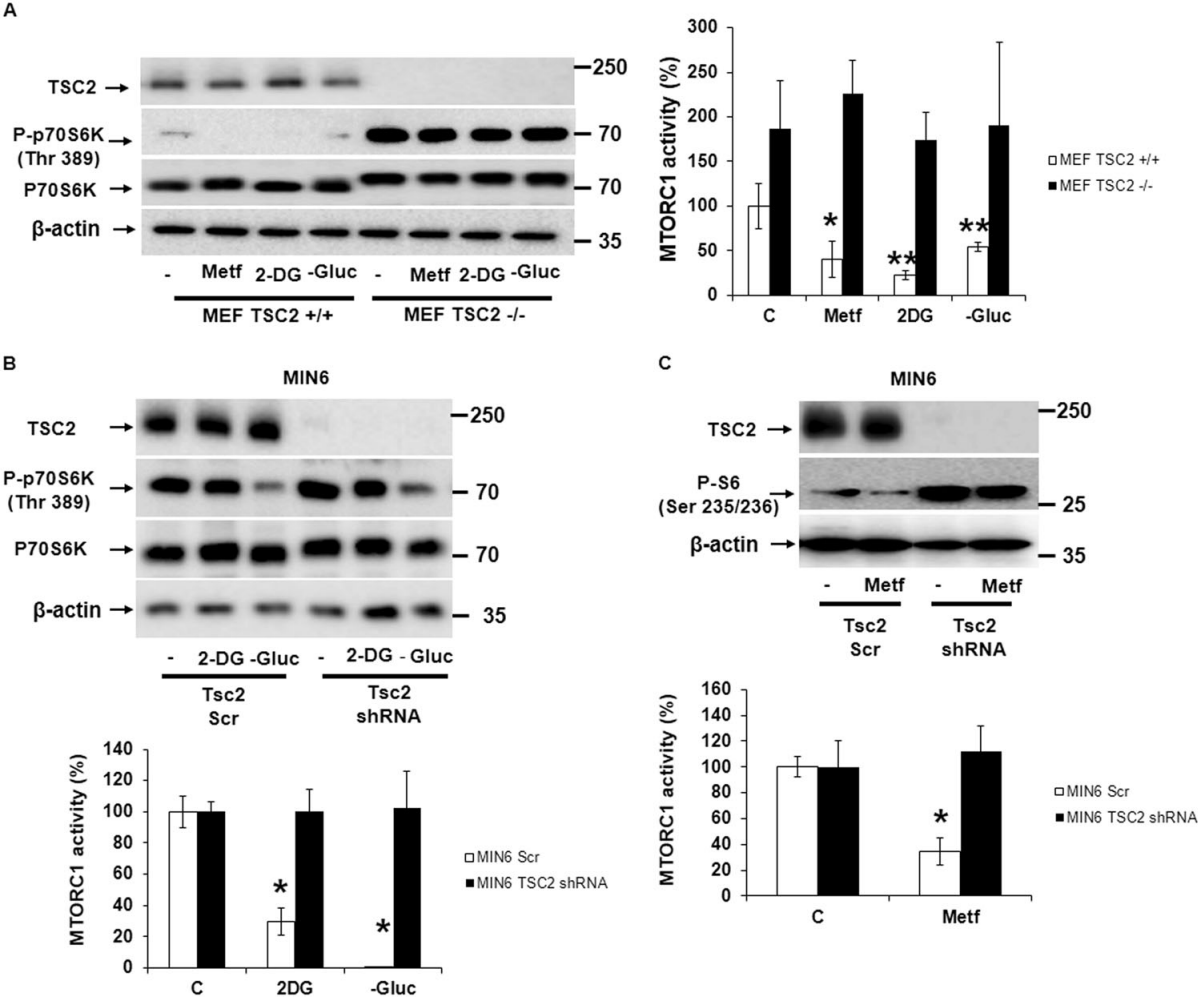
TSC2 plays a key role in the down regulation of MTORC1 in response to glucose energetic stress. **a** Western blot analysis (*n* = 3) of MEF TSC2+/+ and TSC2 −/− cells challenged to different stimuli for 3 h with its corresponding quantification and statistical analysis. The mTORC1 activity (%) represents the percentage of change in P-p70 (Thr 389)/p/70 ratio. *P < 0.05 and **P < 0.01 indicates the comparison between control with either metformin, 2DG or glucose deprivation in MEF TSC2+/+ cells. **b** Western blot analysis (*n* = 3) of MIN6 Scr and TSC2 shRNA cells stimulated with either 2DG or glucose deprivation for 3 h. *P < 0.05, *t-*test (two-tailed, unpaired), comparing control with either 2DG or glucose deprivation in MIN6 scrambled (Scr) cells. **c** Western blot analysis (*n* = 3) of MIN6 Scr and TSC2 shRNA cells stimulated with metformin for 3 h. *P < 0.05, *t*-test (two-tailed, unpaired), is the value corresponding to the comparison between control and metformin treatment in MIN6 Scr cells

Since TSC2 is a key regulator of MTORC1 signaling and its protective role in the maintenance of cell viability under energy stress is known^18^, we challenged both MEF TSC2+/+ and MEF TSC2 −/− cells to different energy stressors (metformin, 2DG, and glucose deprivation). After treatment, we observed a reduction in MTORC1 signaling in MEF TSC2+/+ cells with all stimulants. In contrast, MEF TSC2 −/− cells were refractory to this effect on MTORC1 signaling (Fig. 4a). Similar results were observed when we compared MIN6 Scr and TSC2 shRNA on MTORC1 signaling (Fig. 4b, c). When we knocked-down TSC2 in MIN6 (Supplementary Fig. 4A) and in MEF TSC2 −/− (Supplementary Fig. 4B), there was a reduction in cell viability upon addition of the energetic stressors in both cell types. In addition, there was a recruitment of TSC2 to the lysosome after incubation with either metformin or 2DG, used as a positive control in MEF TSC2+/+ cells (Supplementary Fig. 5A). Similar results were observed when we compared MIN6 Scr to TSC2 shRNA on MTORC1 signaling (Supplementary Fig. 5B). Interestingly, glucose itself was able to localize TSC2 to the membrane or the lysosome in MIN6 cells (Fig. 4e). These results indicate that TSC2 is a crucial element in the inhibition of MTORC1 in response to energetic stress in both MIN6 and in MEF cells.

### Autophagy protects pancreatic beta cells from cell death-induced energetic stress

**Fig. 5:**
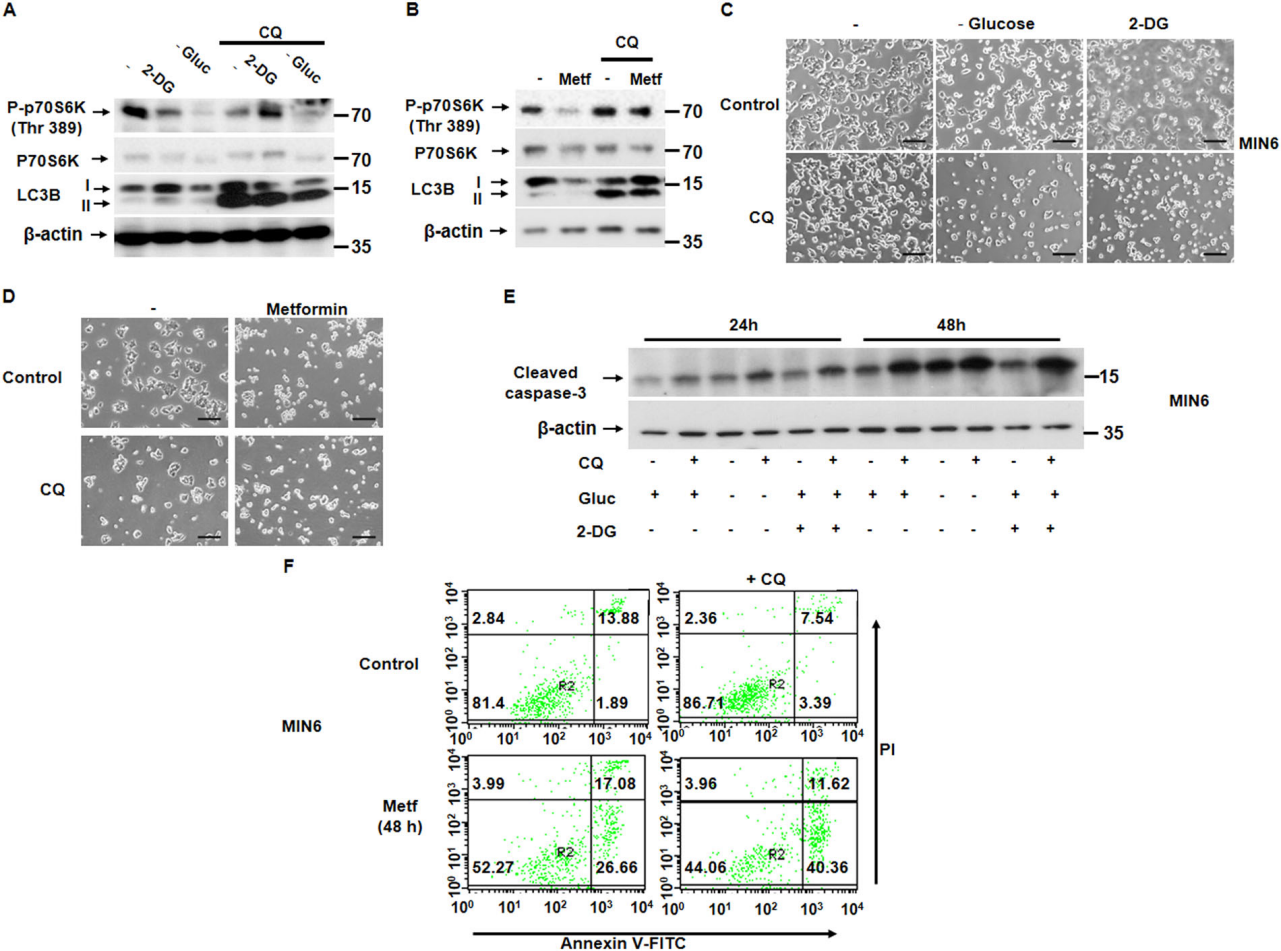
Autophagy plays a protective role in the glucose energetic stress. **a**, **b** Western blot analysis (*n* = 3) of MIN6 cells with and without CQ at 20 nM and in the presence or absence of either 2DG at 25 mM or glucose deprivation (**a**) or metformin 2 mM (**b**) for 24 h. **c**, **d** Representative phase contrast imaging in MIN6 cells treated with either 2DG or glucose deprivation (**c**) or metformin (**d**) in the presence and in the absence of CQ at 20 nM for 48 h. Scale bar, 40 µm. **e** Western blot analysis of MIN6 cells showing cleaved caspase-3 levels in the presence or not of CQ (20 nM) for either 24 h or 48 h. **f** AnnexinV-PI analysis of MIN6 cells exposed to metformin in the presence or in the absence of CQ

Autophagy has been proposed as a protective mechanism in different cell lines, including pancreatic β cells^19^. We decided to subject MIN6 cells to the energetic stressors used before in the presence or the absence of chloroquine (CQ), in order to inhibit autophagy. When we pretreated the cells with CQ in the presence of either 2DG, metformin, or under glucose deprivation, we observed a decrease in MTORC1 (Figs. 5a, b) and a further reduction in cell viability compared with the drugs used alone (Fig. 5c, d). In this regard, when MIN6 were either glucose deprived or treated with 2DG in the presence of CQ, a higher level of cleaved caspase-3 was detected (Fig. 5e). Also, an increase in the percentage of apoptotic cells was also observed after the co-treatment of CQ with metformin compared with the treatment alone (Fig. 5f). These data point to a protective role of autophagy in response to energy deprivation in pancreatic β cells, given that autophagy inhibition by CQ shows a clear reduction in cell survival.

### The phospho-mimetic BAD S155D is an essential regulator of MTORC1 signaling and cell survival under energetic stress in pancreatic β cells

**Fig. 6:**
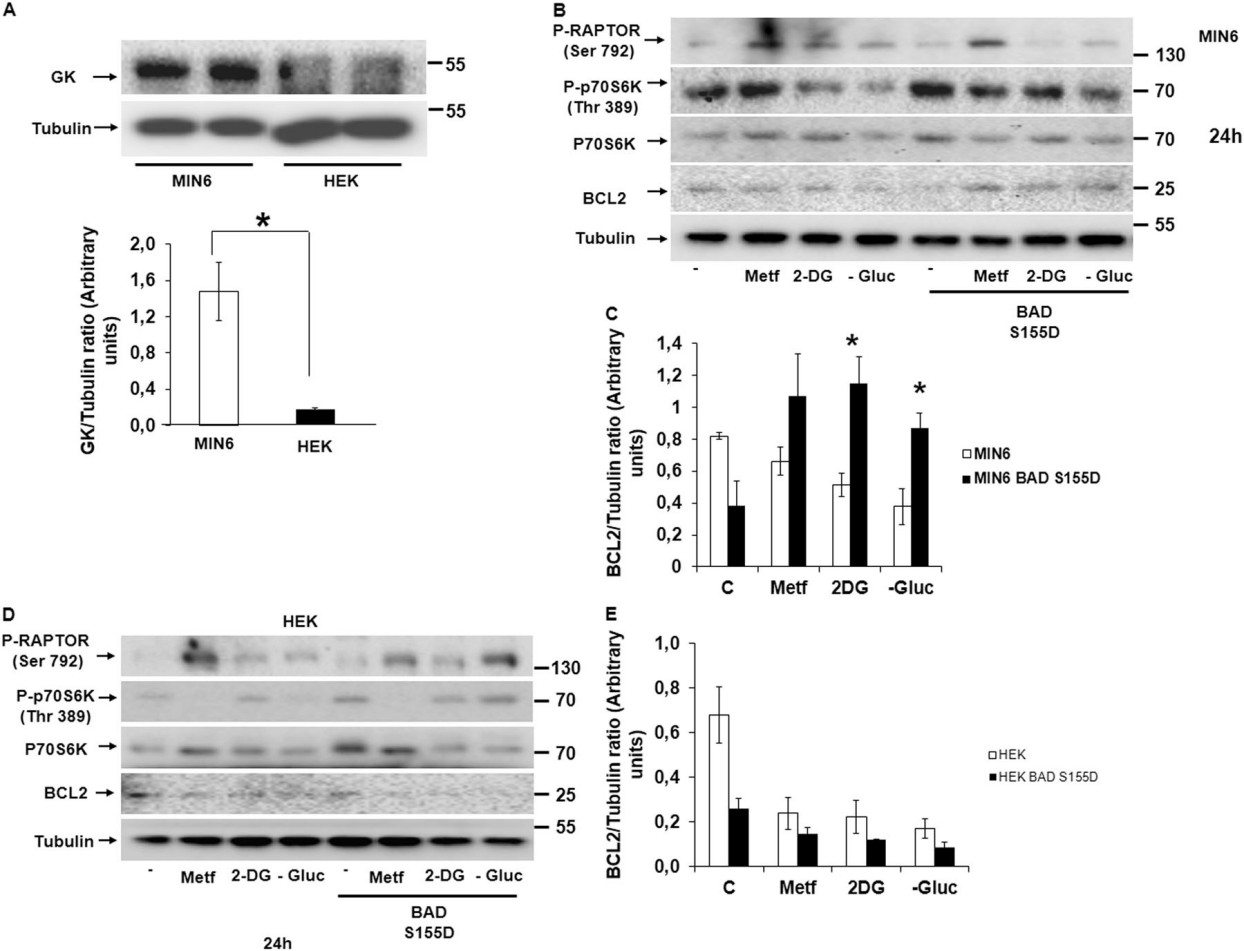
GK is essential in the control of pancreatic β cell viability in response to energy stress. **a** Representative blots showing the comparative endogenous levels of GK protein comparing MIN6 and HEK cells. Data are presented as mean ± SD. *P < 0.05, *t*-test (two-tailed, unpaired), represents the comparison of GK/tubulin ratios in MIN6 with HEK cells. **b** Representative experiment (*n* = 2) in MIN6 with or without the transfection of the phospho-mimetic mutant form of BAD (BADS155D) after the treatment with the different energetic stressor for 24 h. **c** BCL2/tubulin ratios are represented as mean ± SD. *P < 0.05, *t-*test (two-tailed, unpaired), and compares the value of either 2DG or glucose deprivation between MIN6 with or without the transfection with the construct BAD S155D. **d** Representative experiment in HEK cells (*n* = 2) with or without the transfection of the phospho-mimetic mutant form of BAD (BADS155D) after the treatment with the energetic stressors for 24 h. **e** Data are presented as mean ± SD. *P < 0.05

Pancreatic β cells and hepatocytes are the two main cells that present the enzyme hexokinase-IV (GK) and not hexokinase-II (HK-II) as the first enzyme in the glycolysis. Therefore, we first tested the existence of GK in MIN6 and not in HEK cells, showing an intense band corresponding to GK protein exclusively in MIN6 cells (Fig. 6a). It is known that GK can be phosphorylated in multiple residues in response to growth factors, including serine 155. When BAD is phosphorylated at Ser 155 it can associate with GK, mediating a protective effect^10,20,21^. If BAD S155D is transfected to cells without GK activity, such as HEK cells, these would be protected from energetic stress, indicating that GK is not essential for cell survival and that the protection comes exclusively from its dissociation from BCL2 and BcLXL. However, if cell survival only occurs in pancreatic β cells after the transfection of BAD S155D in response to energetic stress, this indicates that the association of GK with the mutant form of BAD is essential in the maintenance of cell viability. After BAD S155D transfection for 24 h, we stimulated the cells with the different compounds. There was a decrease in the induction of AMPK signaling in response to the different energetic stressors in MIN6 transfected with BAD S155D, indicating alleviation in the energetic stress response (Fig. 6b). In contrast, in HEK cells, we did not observe any modulation in the transfected cells in neither MTORC1 signaling nor AMPK regulation (Fig. 6c). More importantly, only in MIN6 transfected cells, there was a statistically significant increase in the anti-apoptotic protein BCL2 after the addition of the different energetic stressors (Fig. 6b–d).

## Discussion

All cells must adapt to the changes in the environmental milieu, and this is especially relevant in pancreatic β cells since they have to coordinate insulin secretion with glucose and amino-acid levels, the two main insulin secretagogues^22^. There is a great amount of information available on how amino-acid levels are sensed and the different protein complexes involved in the regulation of MTORC1, as reviewed previously^23^. However, much less is known on the molecular mechanisms modulating the absence of glucose or other energetic stressors such as metformin, 2DG, or glucose deprivation itself. In this report, we demonstrate that pancreatic β cells (MIN6 and INS1E cells) and hepatocytes are especially prone to cell death in response to glucose energy deprivation. Only MIN6 cells were capable to down-regulate MTORC1 signaling when glucose levels dropped from 25 to 5 mM. More importantly, beyond this glucose concentration (1, and 0.5 mM of glucose), we observed an activation of caspase-3 protein levels in MIN6 cells and not in MEF cells. These results clearly indicate that pancreatic β cells sense glucose deprivation in a more sensitive way than other cell types do, making them more susceptible to its withdrawal. This observation is coordinated with the decreased cellular viability observed in these cells exposed to glucose deprivation. In the same way, previous reports show that pancreatic β cells are highly susceptible to apoptosis or dysfunction when a chronic activation of AMPK occurs^11,16,17^. Constitutive activation of AMPK under normal conditions increased the cleaved form of caspase-3, indicative of apoptosis induction^24^. In said study, the authors suggest that the increased susceptibility, at least under glucose deprivation, is due to the inhibition of insulin secretion when there is a low energy status of the cell^25^. Metformin, which is the first line of treatment for T2DM, has been described to cause pancreatic β cell death by apoptosis. This effect could be reversed by the addition of methyl succinate, to bypass metformin blockade of complex I, inducing complex II activity^26^. Our data indicate that metformin decreases cell viability, especially in β cells and additionally at lower doses compared to non-β cells.

The mechanism of activation of AMPK by glucose deprivation is multifactorial. Firstly, glucose deprivation facilitates an increase in both AMP/ATP and ADP/ATP ratios, boosting the kinase activity of AMPK toward its downstream effectors. Secondly, very recently, it has been demonstrated that glucose deprivation can also activate a mechanism independent of variations in the level of adenine nucleotides. One of the glycolytic intermediates, fructose-1,6-bisphosphate (FBP) can be sensed by aldolase. This complex (FBP-aldolase) interacts with the vacuolar-ATPase (v-ATPase) on the surface of the lysosome. The absence of FBP, characteristic of glucose deprivation, alters the interaction between v-ATPase and aldolase and permits the interaction with AMPK-AXIN and v-ATPase, Ragulator and LKB1. This new interaction facilitates the phosphorylation of AMPK at threonine 172 by LKB1^14,15^. This is very interesting since there is a connection between glycolysis and MTORC1 control by the modulation of AMPK in all cells. Although AMPK has been proposed as a master regulator of metabolic homeostasis, it can be activated in both an AMP-dependent and AMP-independent manner, as previously indicated. In addition, there is some hierarchy in the compartmentalization of the AMPK response, depending on the severity of the energetic stress^27^. For instance, MEF cells subjected to low glucose (5 mM or below) showed no change in either AMP levels or cytosolic AMPK activation, being only active in the lysosomal fraction. Our data fit perfectly with these results and indicate that the severity of the energetic stress produced in pancreatic β cells in response to any of the energetic stressors used in this work is much more pronounced than in MEF or HEK cells. It is likely that other compartments apart from the lysosomal one are involved in these responses. Very interestingly, recent data indicate that the KICSTOR complex, which is involved in the regulation of MTORC1 in response to amino-acid levels, could be involved in glucose sensing^28^.

A link between metabolism and cell survival has been determined specifically in pancreatic β cells and hepatocytes, mediated by the interaction of GK and the pro-apoptotic protein BAD, on the outer membrane of mitochondria^29^. Considering that the interaction between phospho-BAD (at Ser155) and GK promotes cell survival under different stress situations^10,21^, we think that, this interaction could mediate cell survival only in GK-positive cells. Our results indicate that overexpression of the phosphor-mimetic mutant form of BAD enhances a pro-survival environment only revealed in β cells. This protection could be mediated by the increase in BCL2 protein levels, which was observed only in pancreatic β cells. These data suggest that there is a link between the mutant form of BAD and GK, facilitating protection from the pro-apoptotic effect of the different energetic stress treatments. However, HK has been defined as a key enzyme for autophagy activation in response to glucose withdrawal. In fact, HK and MTORC1 interact directly, decreasing MTORC1 activity in a glucose starvation-dependent manner. Therefore, HK seems to exert a protective role in the cell types that presents this enzyme by the induction of autophagy under energy deprivation^30^.

Apart from GK and AMPK, other proteins regulate MTORC1 under nutrient deprivation situations, such as TSC2. This regulator has been involved in the control of MTORC1 in response to different stimuli, including amino-acid deprivation, 2DG stimulation or a hyper-osmotic stress by the translocation of TSC2 to the membrane of the lysosome^5–7,31^. Our present findings show the key role of TSC2 in the control of MTORC1 regulation in response to energetic deprivation in both pancreatic β cells and non-pancreatic β cells. In this context, the implication of TSC in the maintenance of cell survival under glucose deprivation has been analyzed before in MEF^18^, but it was completely unknown in pancreatic β cells. Therefore, this glucose addiction that TSC mutant cells present towards glucose is due to its impairment in the down-regulation of MTORC1 signaling^32^. Furthermore, both deleting TSC2 in MEF cells and knocking it down in β cells abrogates MTORC1’s ability to be down-regulated under energetic stress. The data obtained in this paper corroborate that TSC2 is an essential element involved in the down-regulation of MTORC1 in response to energetic stress in both pancreatic β cells and non-pancreatic β cells. MTORC1 activity has to be maintained and thus modulation has to be adapted to each situation. This is why MTORC1 hyperactivation is not beneficial since it does not allow a correct autophagic flux and the elimination of damaged mitochondria^33^. In contrast, a down-regulation of MTORC1 can be deleterious for pancreatic β cells as well, since it alters homeostasis and insulin processing^34^, and it impairs its expansion in response to either a high fat diet^35^ or an ER stress^36^.

**Fig. 7:**
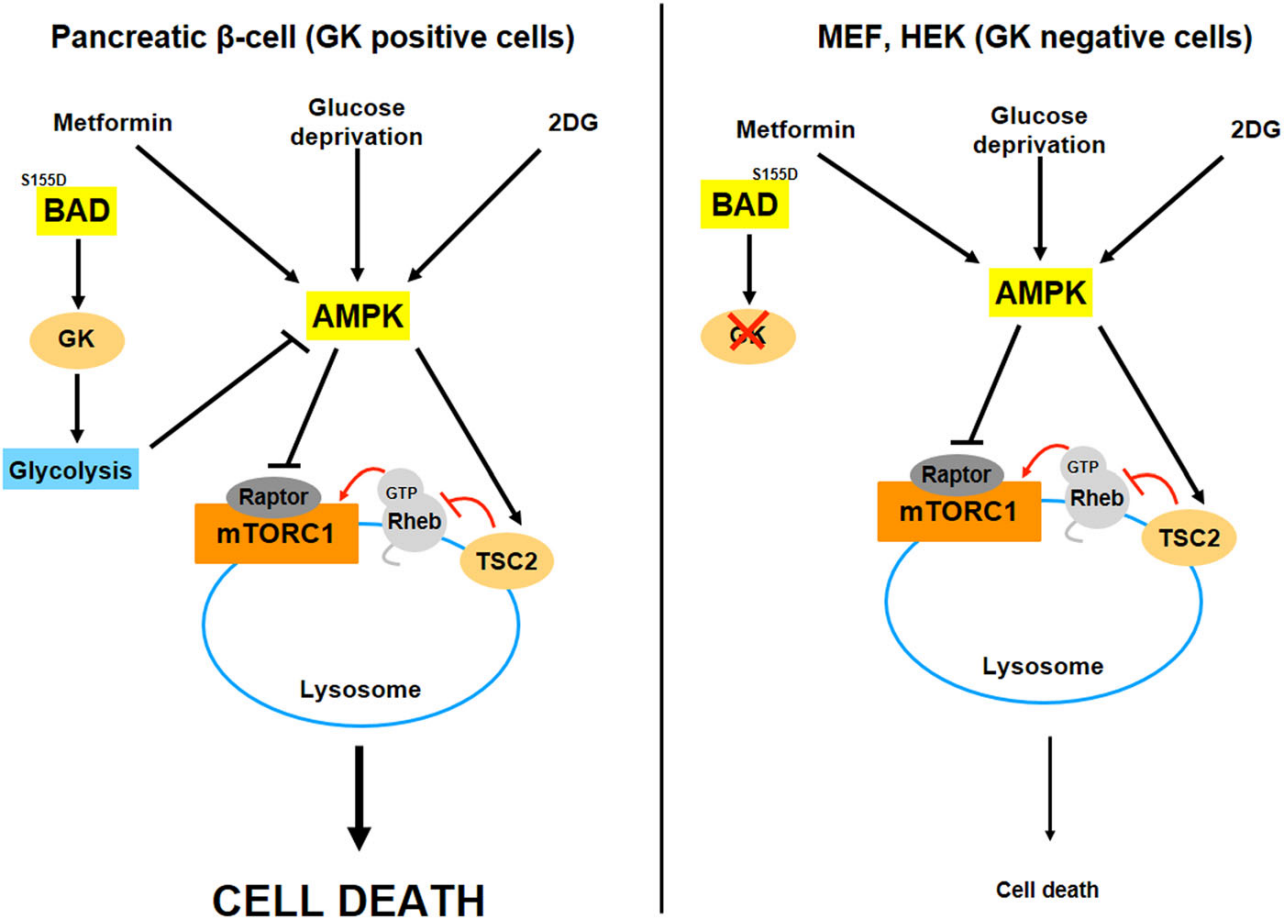
Role of the glucokinase-dependent and independent mechanisms converging on MTORC1 signaling regulation comparing pancreatic and non-pancreatic β cells

In summary, pancreatic β cells are more susceptible to energetic stress compared to other cell types, being essential for adaptation to energy deprivation; TSC2 and GK. The use of different strategies affecting these signaling pathways, such as GK activators or a preserved TSC2 activity, could be beneficial for the maintenance of pancreatic β cells for longer periods of time, avoiding its loss in pathologies such as type 2 diabetes mellitus. Figure 7 portrays the most important conclusions derived from the present paper.

## Materials and methods

### Antibodies and reagents

The following antibodies were obtained from Cell Signaling Technology (Beverly, MA): anti-MAP1LC3/LC3 (#4108), anti-RPS6KB/p70S6K (#9202), anti-phospho-RPS6KB/p70S6K (Thr389) (#9205), anti-TSC2 (D93F12, #4308), anti-phospho-RAPTOR (Ser 792) (#2083), anti-RAPTOR (#2280), anti-cleaved caspase-3 (#9661), anti-phospho-ACC (Ser 79) (#3661). Anti-phospho-S6 was obtained from Thermo-Scientific (MA5-15140). Anti-LAMP2 (#ab13524) antibody was obtained from Abcam. The anti-glucokinase (GK) (sc-7908) was obtained from Santa Cruz. From Sigma-Aldrich: anti-ACTB/β-actin (A5316). Chloroquine C6628 and propidium iodide (P4170) were from Sigma-Aldrich; 2-deoxy-glucose (2DG) (D8375) was from Merck; and geneticin (G418) (sc-29065) and hygromycin (sc-29067) were from Santa Cruz.

### Cell culture

Rat insulinoma INS-IE cells were kindly provided by P. Maechler (Université de Genève). INS1E were cultured in 10% FBS RPMI 1640 supplemented with 1 mM sodium pyruvate, 10 mM HEPES and 50 μM 2-mercaptoethanol^37^. Mouse insulinoma MIN6 cells were cultured in 15% FBS DMEM supplemented with 50 µM 2-mercaptoethanol. All the MEF cell lines were cultured in 10% FBS DMEM. For the experiments, cells were seeded and the next day, cells were changed with fresh complete medium for 2 h, after which they were stimulated with either metformin at 2 mM or 2DG at 25 mM for different periods of time. In the case of the glucose deprivation experiments, cells were seeded as mentioned before and the next day, cells were changed with complete medium without glucose for the different periods of time. Regular testing for mycoplasma detection was performed. In some experiments we co-treated the cells with CQ and with the corresponding stimuli for 24 h.

### Lentivirus and retrovirus production for cell infection

HEK293T cells were co-transfected using lipofectamine 2000 with the lentiviral packaging plasmids pMD2.G (Addgene 12259) and psPAX2 (Addgene, 12260) with either pLKO-1-*Scrambled*-neo or pLKO.1-*Tsc2*-neo (Addgene, 24150).

### Transfection with the phosphomimetic mutant of BAD (BADS155D)

MIN6 and HEK cells were cultured in six-multiwell dishes at sub-confluency. The following day transfection was completed using TRANS-IT-293 reagent according to manufacturer´s instructions. A mixture of DNA (300 ng plasmid), TRANS-IT-293 reagent (6 µl/µg plasmid) and OPTIMEM was incubated for 20 min. Meanwhile, the medium was changed for fresh medium and after the incubation, the transfection mixture was added. After 24 h and without changing the medium, cells were stimulated for 24 h. The next day the dishes were scraped with the supernatant and centrifuged at 1200 rpm for 5 min. The pellet was washed in PBS and centrifuged again. The pellet obtained after centrifugation at 12,000 rpm for 20 min was resuspended in lysis buffer to obtain the crude protein extract.

### Flow cytometry

For cell cycle analysis, both trypsinised adherent and non-adherent cells were collected by centrifugation and then were fixed with cold ethanol (70% v/v). After that, the cells were washed, resuspended in PBS, and incubated with RNase for 30 min at 37 °C. After addition of 0.05% propidium iodide (w/v), cellular DNA content was quantified by flow cytometry. Annexin V-FITC Apoptosis Detection Kit (Immunostep, ANNEXINVKIT) assays were carried following the instructions from the manufacturer.

### Western blotting

After the different treatments, cells were washed twice with PBS and lysed for protein extraction according to standard procedures. Protein concentration determination was achieved by the Bradford dye method, using the Bio-Rad (Hercules, CA) reagent and BSA as standard. Equal amounts of protein (15–20 µg) were submitted to electrophoresis and after SDS–PAGE gels were transferred to Immobilon P PVDF membranes (Merck–Millipore). Then, membranes were blocked with 5% non-fat dried milk and incubated overnight with antibodies at 4 °C. The corresponding bands were visualized using the ECL Western blotting protocol (GE Healthcare, Little Chalfont, UK). All the blots presented in the paper are non-saturated signals being at the linear range of exposition with the following exceptions: in the blots from the Fig. 3a we present a long exposure to compare the differential effect of either metformin or glucose deprivation on phospho-p70S6K (Thr 389) and p70S6K in MIN6 and MEF cells. Similarly, in Fig. 4a–c, we show a long exposure of phospho-P70S6K (Thr 389) or phospho-S6 (Ser 235/236) to see the hyperactivation of MTORC1 signaling and the inhibitory effect of the energetic stressors on MTORC1 signaling.

### Immunofluorescence and co-localization analysis

Cells were grown on glass coverslips and fixed using paraformaldehyde 4% for 15 min, permeabilized in PBS with 0.5% Triton X-100 for 10 min and then blocked with (3% BSA, 0,1% Tween 20 in PBS) for 1 h. Cells were incubated o/n at 4°C with primary antibodies (1:100 in blocking solution). After this incubation, coverslips were incubated with the corresponding secondary antibodies, at a dilution of 1:100 for 1 h. For co-localization analysis, images were processed with Coloc2 (http://fiji.sc/Fiji). The threshold was obtained automatically using Costes automatic threshold and the thresholded Manders´ coefficient M1 was determined^38^.

### Violet crystal assay

INS1E, MIN6 and hepatocytes cell lines were seeded in 12-well plates at a density of 5000 cells/cm^2^ in either DMEM supplemented with FBS 10% in the case of MIN6 and hepatocytes or in RPMI supplemented with FBS 15% in the case of INS1E. After 24 h, cells were treated at different concentrations for 48 h. After that, cells were washed twice with cold PBS and then stained with 0.2% violet crystal (w/v) in 2% ethanol (v/v) for 10 min. Plates were rinsed with ddH2O, dried, and after addition of 1% sodium dodecyl sulfate (w/v), absorbance at 560 nm was determined for each time point.

### Statistics

Statistically significant differences between mean values were determined using the unpaired Student *t*-test in the Graphpad statistical analysis software package. Differences were considered statistically significant at *P* < 0.05, *P < 0.05, **P < 0.01, ***P < 0.001, ****P < 0.0001, while n.s indicates no statistical significance. For colocalization of TSC2-LAMP2 we performed an ANOVA study using the Newman–Keuls analysis as post hoc.

## Supplementary information

Supplementary Figures

Supplementary Figure Legends
