# Supplementary material for: Essential role of glucokinase in the protection of pancreatic β cells to the glucose energetic status
Source: Cell Death Discov. 2019 Sep 30;5:138. doi: 10.1038/s41420-019-0219-x (PMC6769003; doi:10.1038/s41420-019-0219-x)
Supplement: Supplementary file 1 — Supplementary Figures [file 41420_2019_219_MOESM1_ESM.pptx]

## Slide 1
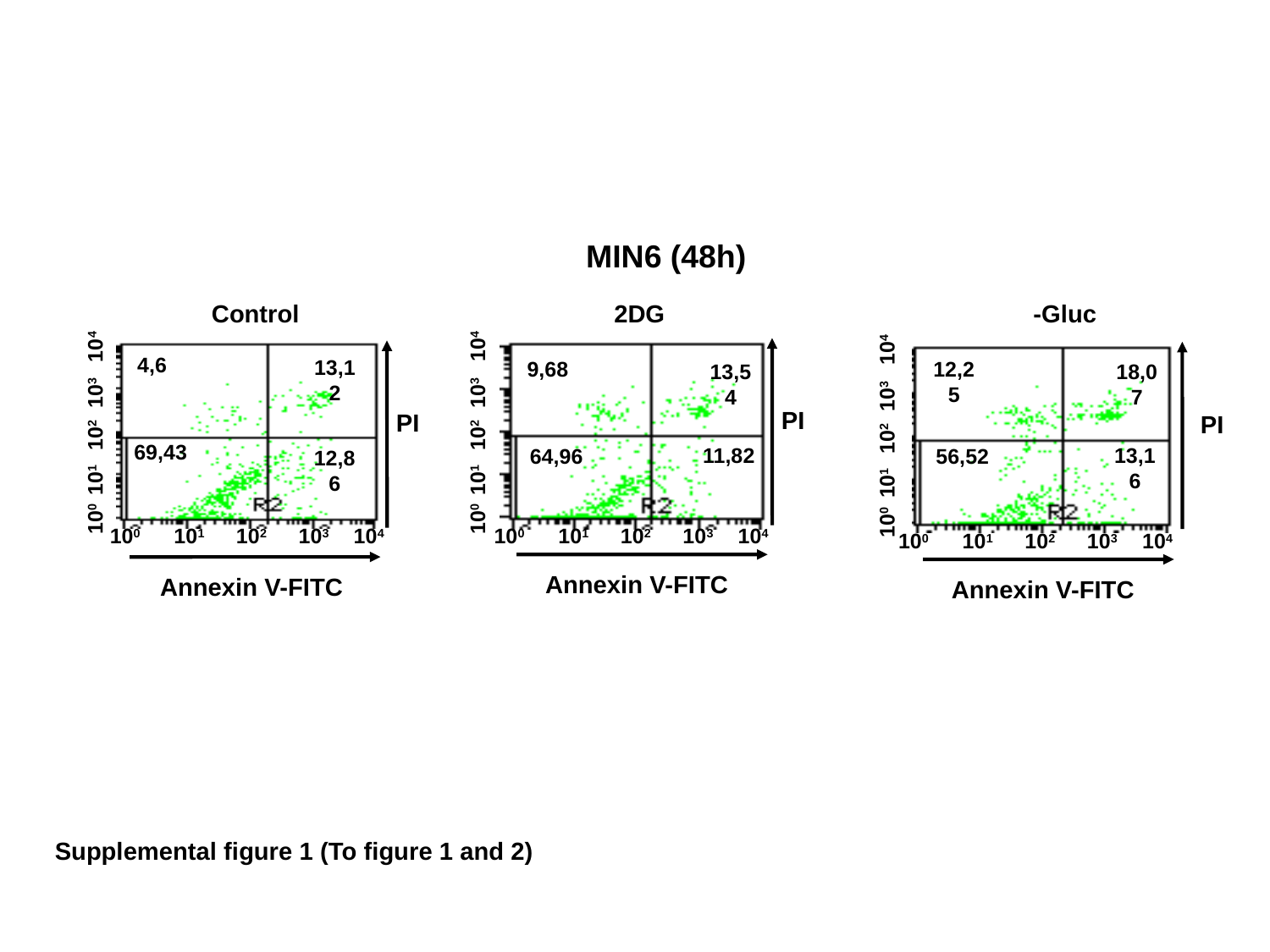

MIN6 (48h)
Control
2DG
-Gluc
104
104
104
4,6
13,12
12,25
9,68
18,07
13,54
103
103
103
PI
PI
PI
102
102
102
69,43
13,16
11,82
56,52
64,96
12,86
101
101
101
100
100
100
100
101
102
103
104
100
101
102
103
104
100
101
102
103
104
Annexin V-FITC
Annexin V-FITC
Annexin V-FITC
Supplemental figure 1 (To figure 1 and 2)

## Slide 2
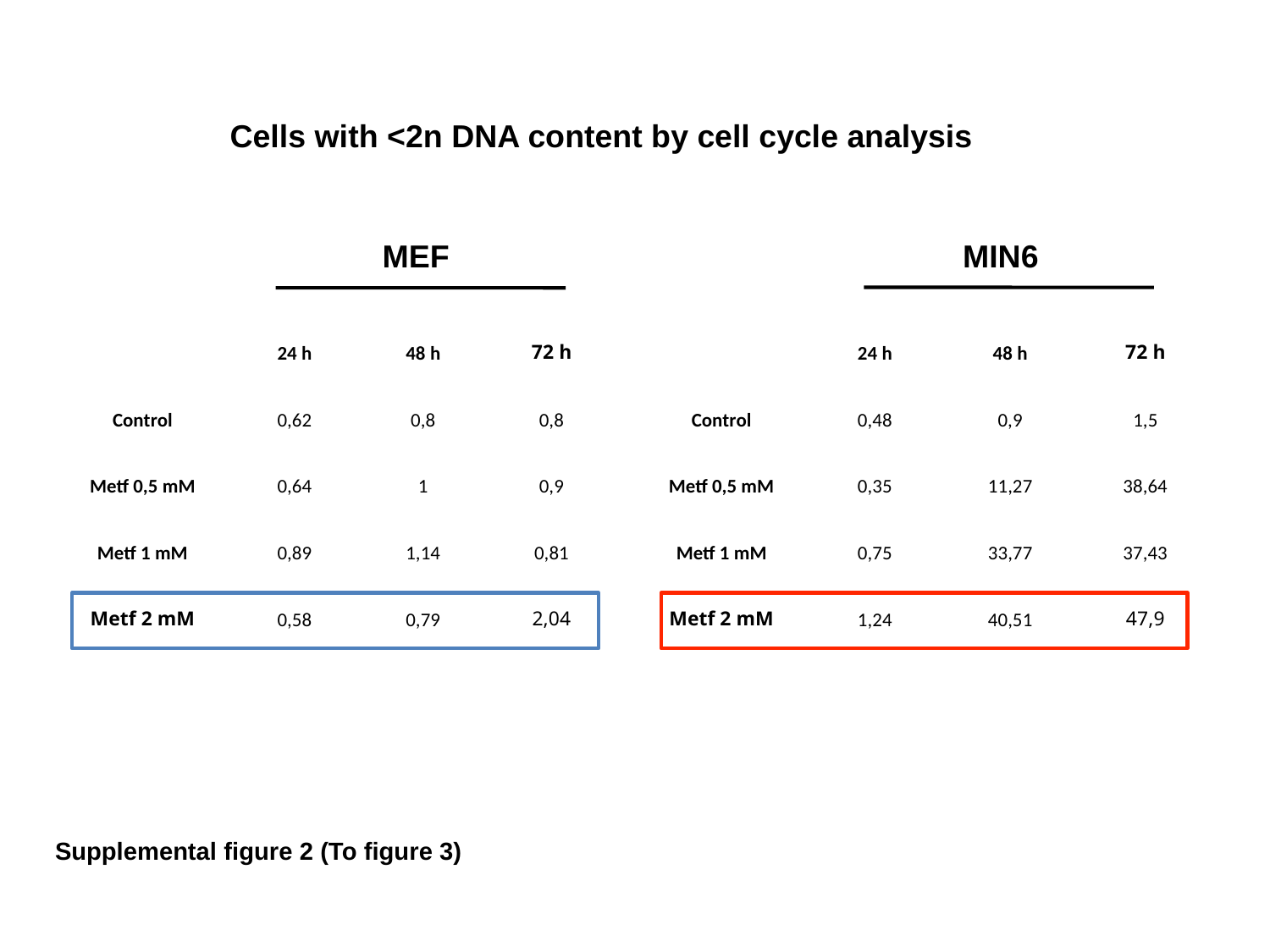

Cells with <2n DNA content by cell cycle analysis
MIN6
MEF
| | 24 h | 48 h | 72 h |
| --- | --- | --- | --- |
| Control | 0,62 | 0,8 | 0,8 |
| Metf 0,5 mM | 0,64 | 1 | 0,9 |
| Metf 1 mM | 0,89 | 1,14 | 0,81 |
| Metf 2 mM | 0,58 | 0,79 | 2,04 |
| | 24 h | 48 h | 72 h |
| --- | --- | --- | --- |
| Control | 0,48 | 0,9 | 1,5 |
| Metf 0,5 mM | 0,35 | 11,27 | 38,64 |
| Metf 1 mM | 0,75 | 33,77 | 37,43 |
| Metf 2 mM | 1,24 | 40,51 | 47,9 |
Supplemental figure 2 (To figure 3)

## Slide 3
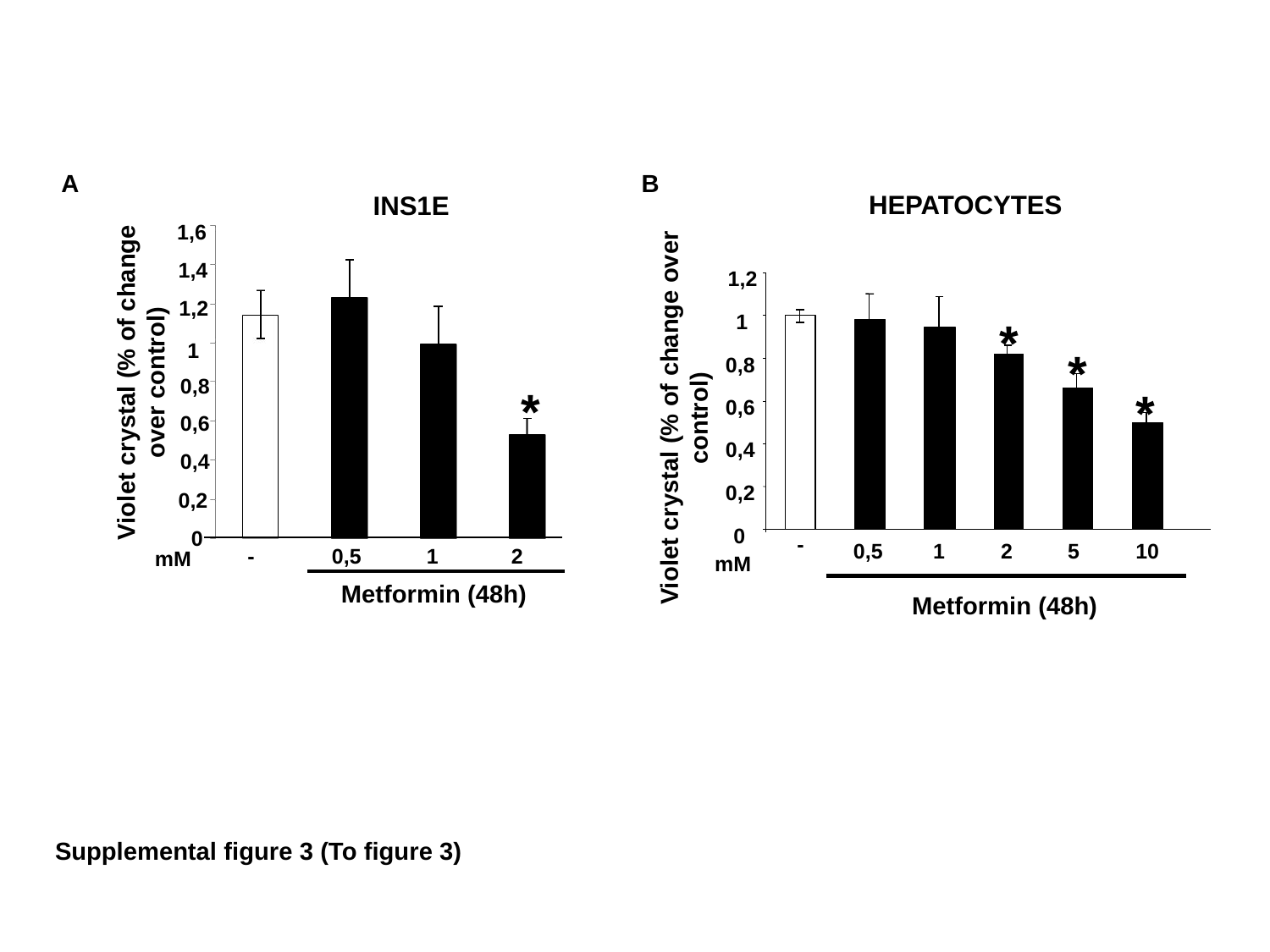

A
B
HEPATOCYTES
1,2
*
1
*
0,8
*
Violet crystal (% of change over control)
0,6
0,4
0,2
0
-
0,5
1
2
5
10
mM
Metformin (48h)
INS1E
1,6
1,4
1,2
1
Violet crystal (% of change over control)
0,8
*
0,6
0,4
0,2
0
-
0,5
1
2
mM
Metformin (48h)
Supplemental figure 3 (To figure 3)

## Slide 4
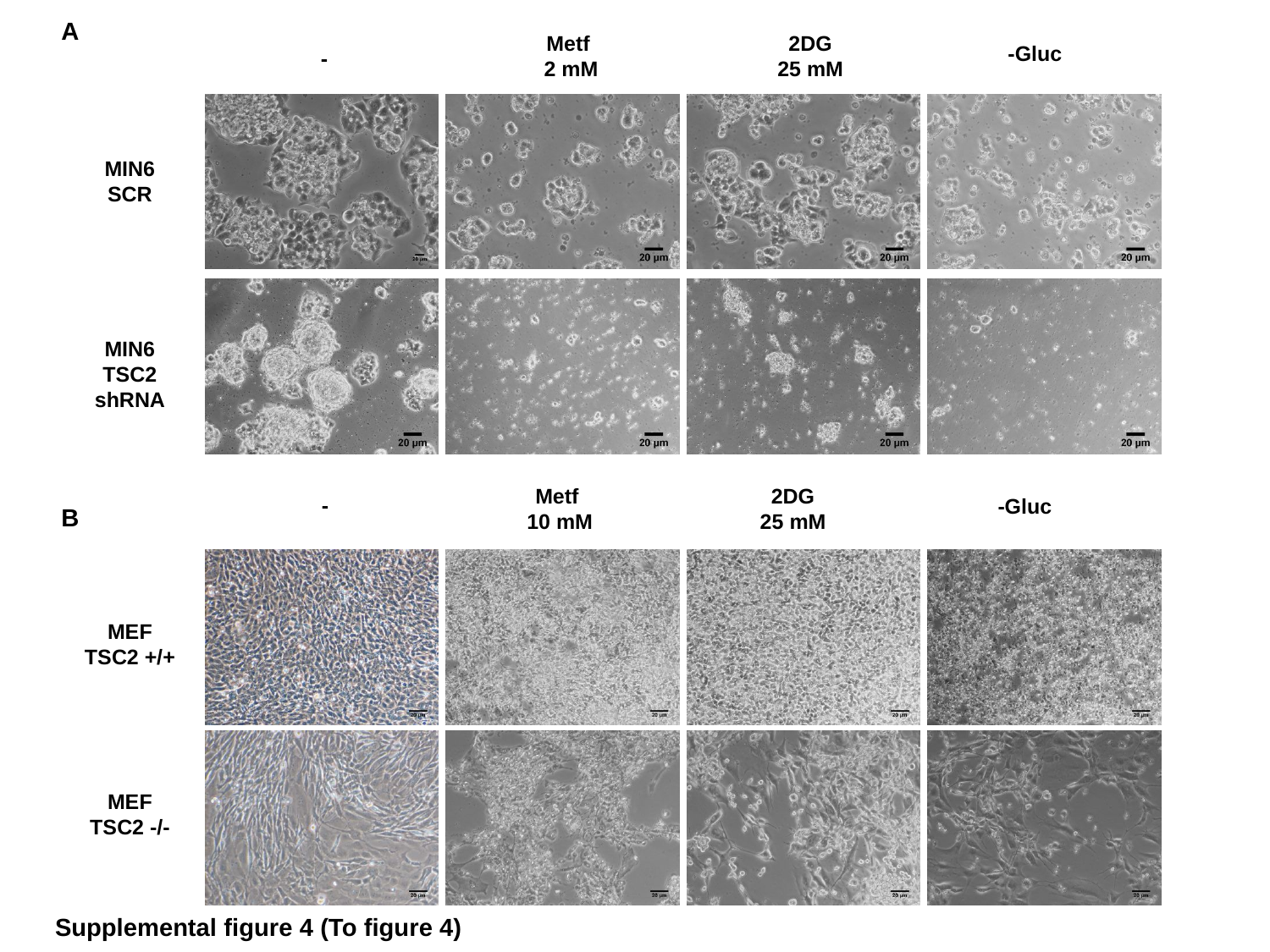

A
Metf
2 mM
2DG
25 mM
-Gluc
-
MIN6
SCR
MIN6
TSC2
shRNA
Metf
10 mM
2DG
25 mM
-
-Gluc
B
MEF
TSC2 +/+
MEF
TSC2 -/-
Supplemental figure 4 (To figure 4)

## Slide 5
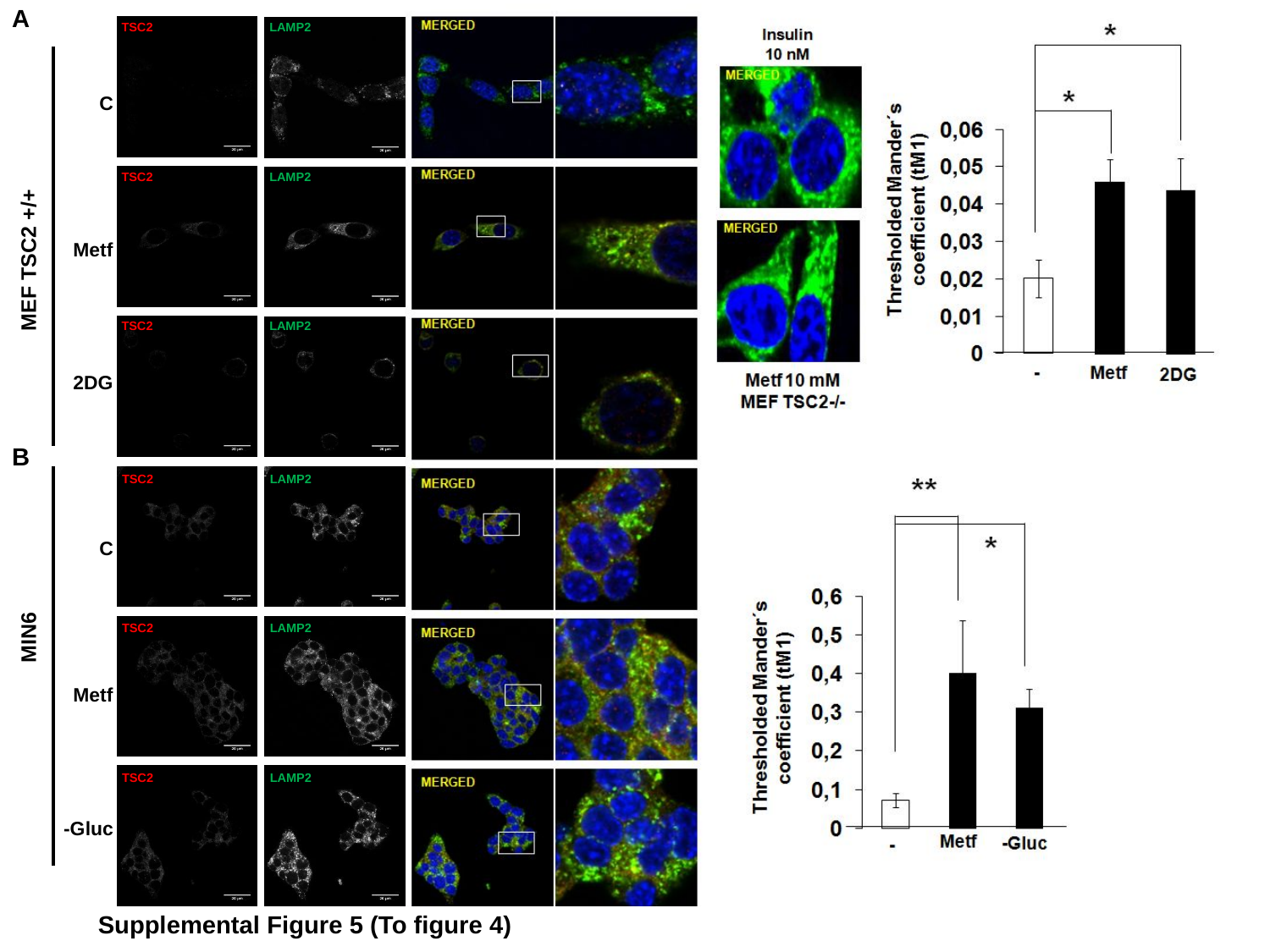

A
TSC2
LAMP2
C
TSC2
LAMP2
Metf
MEF TSC2 +/+
TSC2
LAMP2
2DG
B
TSC2
LAMP2
C
TSC2
LAMP2
MIN6
Metf
TSC2
LAMP2
-Gluc
Supplemental Figure 5 (To figure 4)
