# Supplementary material for: Essential role of glucokinase in the protection of pancreatic β cells to the glucose energetic status
Source: Cell Death Discov. 2019 Sep 30;5:138. doi: 10.1038/s41420-019-0219-x (PMC6769003; doi:10.1038/s41420-019-0219-x)
Supplement: Supplementary file 2 — Supplementary Figure Legends [file 41420_2019_219_MOESM2_ESM.docx]

**Supplemental Figure 1.** AnnexinV-PI analysis of MIN6 cells exposed to either 2DG or glucose deprivation for 48h.

**Supplemental Figure 2**. Comparative dose-response (from no metformin to 2 mM) of MEF and MIN6 cells after treatment with the drug for 24h, 48h and 72h using flow cytometry. The numbers indicate the percentage of cells in haplodiploid phase.

**Supplemental Figure 3.** Cell viability after metformin treatment for 48h in both INS1E (A) and hepatocytes (B). The percentage of change over control represents the mean+/- SD. * P<0.05.

**Supplemental Figure 4. A.** Representative images from MIN6 SCR or with a knock-down of TSC2 exposed to the different energetic stressors for 48h. **B**. Representative images obtained from MEF TSC2+/+ or TSC2-/- exposed to different energetic stressors for 48 hours. Scale bar, 20 µm.

**Supplemental Figure 5**. Co-localization analysis using immunofluorescence analysis of MEF TSC2+/+ cells after the treatment with either metformin or EBSS. MEF TSC2 +/+ treated with insulin 10 nM and MEF TSC2 -/- cells treated with metformin were used as negative controls. EBSS stands for Earle’s balanced salt solution. * P<0.05. Scale bar, 20 µm.
